# (−)-Epigallocatechin-3-Gallate (EGCG) Enhances Osteogenic Differentiation of Human Bone Marrow Mesenchymal Stem Cells

**DOI:** 10.3390/molecules23123221

**Published:** 2018-12-06

**Authors:** Sung-Yen Lin, Lin Kang, Chau-Zen Wang, Han Hsiang Huang, Tsung-Lin Cheng, Hsuan-Ti Huang, Mon-Juan Lee, Yi-Shan Lin, Mei-Ling Ho, Gwo-Jaw Wang, Chung-Hwan Chen

**Affiliations:** 1 Orthopaedic Research Center, Kaohsiung Medical University, Kaohsiung 80701, Taiwan; tony8501031@gmail.com (S.-Y.L.); czwang@kmu.edu.tw (C.-Z.W.); junglecc@gmail.com (T.-L.C.); hthuang@kmu.edu.tw (H.-T.H.); 327lin@gmail.com (Y.-S.L.); homelin@cc.kmu.edu.tw (M.-L.H.); gwojaw@cc.kmu.edu.tw (G.-J.W.); 2 Department of Orthopedics, Kaohsiung Medical University Hospital, Kaohsiung Medical University, Kaohsiung 80701, Taiwan; 3 Departments of Orthopedics, College of Medicine, Kaohsiung Medical University, Kaohsiung 80701, Taiwan; 4 Department of Orthopedics, Kaohsiung Municipal Ta-Tung Hospital, Kaohsiung 80415, Taiwan; 5 Graduate Institute of Medicine, Kaohsiung Medical University, Kaohsiung 80701, Taiwan; 6 Department of Obstetrics and Gynecology, National Cheng Kung University Hospital, College of Medicine, National Cheng Kung University, Tainan 70403, Taiwan; kanglin@mail.ncku.edu.tw; 7 Department of Physiology, College of Medicine, Kaohsiung Medical University, Kaohsiung 80701, Taiwan; 8 Department of Medical Research, Kaohsiung Medical University Hospital, Kaohsiung 80701, Taiwan; 9 Department of Veterinary Medicine, National Chiayi University, Chiayi 60054, Taiwan; hhuang@mail.ncyu.edu.tw; 10 Department of Bioscience Technology, Chang Jung Christian University, Tainan 71101, Taiwan; mjlee@mail.cjcu.edu.tw; 11 Innovative Research Center of Medicine, Chang Jung Christian University, Tainan 71101, Taiwan; 12 Department of Marine Biotechnology and Resources, National Sun Yat-sen University, Kaohsiung 80424, Taiwan

**Keywords:** (−)-epigallocatechin-3-gallate (EGCG), antioxidant, human bone marrow mesenchymal stem cells (BMSCs), mineralization, osteogenesis

## Abstract

Osteoporosis is the second most-prevalent epidemiologic disease in the aging population worldwide. Cross-sectional and retrospective evidence indicates that tea consumption can mitigate bone loss and reduce risk of osteoporotic fractures. Tea polyphenols enhance osteoblastogenesis and suppress osteoclastogenesis in vitro. Previously, we showed that (−)-epigallocatechin-3-gallate (EGCG), one of the green tea polyphenols, increased osteogenic differentiation of murine bone marrow mesenchymal stem cells (BMSCs) by increasing the mRNA expression of osteogenesis-related genes, alkaline phosphatase activity and, eventually, mineralization. We also found that EGCG could mitigate bone loss and improve bone microarchitecture in ovariectomy-induced osteopenic rats, as well as enhancing bone defect healing partially via bone morphogenetic protein 2 (BMP2). The present study investigated the effects of EGCG in human BMSCs. We found that EGCG, at concentrations of both 1 and 10 µmol/L, can increase mRNA expression of BMP2, Runx2, alkaline phosphatase (ALP), osteonectin and osteocalcin 48 h after treatment. EGCG increased ALP activity both 7 and 14 days after treatment. Furthermore, EGCG can also enhance mineralization two weeks after treatment. EGCG without antioxidants also can enhance mineralization. In conclusion, EGCG can increase mRNA expression of BMP2 and subsequent osteogenic-related genes including Runx2, ALP, osteonectin and osteocalcin. EGCG further increased ALP activity and mineralization. Loss of antioxidant activity can still enhance mineralization of human BMSCs (hBMSCs).

## 1. Introduction

Osteoporosis, one of the most prevalent diseases in the elderly, is caused by the imbalance between osteoblastic and osteoclastic regulations [1]. Treatment of osteoporosis can be achieved by enhancing bone formation or decreasing bone resorption [1]. Most current medications for osteoporosis inhibit bone resorption, and only teriparatide has an osteogenic effect [2,3]. Therefore, identifying compounds and molecules that stimulate bone formation is important for effective treatment of osteoporosis.

Tea, brewed from dried leaves of the plant *Camellia sinensis*, is one of the most popular beverages in the world, with 3 billion kg consumed annually [4,5]. Green tea is a non-oxidized and non-fermented product containing several polyphenolic components. Most green tea polyphenols are catechins (3,3′,4′,5,7-pentahydroxyflavan). The major catechins in green tea are (−)-epicatechin (EC), (−)-epicatechin gallate (ECG), (−)-epigallocatechin (EGC) and (−)-epigallocatechin-3-gallate (EGCG). Many beneficial effects of catechins depend on their antioxidant and free radical scavenging activities [6,7]. Among these catechins, EGCG is the most potent antioxidant. Despite having numerous reports on the beneficial effects of tea, the osteogenic effects of catechins have been less extensively studied.

Epidemiological studies revealed that post-menopausal habitual tea drinkers have higher bone mineral density (BMD) [8,9] and reduced risk of hip fractures [10,11]. We previously found EGCG, 1 and 10 μmol/L, enhanced osteogenic differentiation of murine bone marrow mesenchymal stem cell by enhancing the mRNA expression of osteogenesis-related genes, alkaline phosphatase (ALP) activity and, eventually, mineralization [12]. To the best of our knowledge, the effects of EGCG on human bone marrow stem cells (hBMSCs) have rarely been reported [13,14,15]. Therefore, we hypothesize that EGCG can induce hBMSCs toward osteogenic differentiation, similar to those in the murine BMSCs. In this study, we examined the osteogenic effects of EGCG in hBMSCs and the molecular mechanism of its effects. We also evaluated whether antioxidant activity of EGCG played an important role in osteogenic effects.

## 2. Results

### 2.1. 3-(4,5-Dimethylthiazol-2-yl)-2,5-diphenyltetrazolium Bromide (MTS) Assay

**Figure 1:**
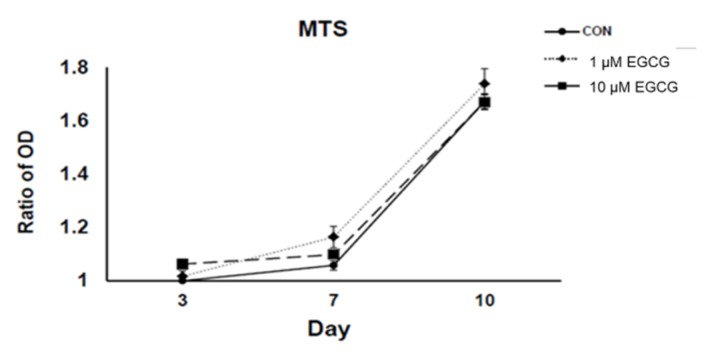
Effects of (−)-epigallocatechin-3-gallate (EGCG) on human bone marrow stem cells (hBMSCs) in 3-(4,5-Dimethylthiazol-2-yl)-2,5-Diphenyltetrazolium Bromide (MTS). There was no significant change in MTS assay and cell cycle after EGCG treatment at 1 and 10 µmol/L for 24 and 48 h. With treatment of EGCG, the viability of hBMSCs was not affected by EGCG at both 1 and 10 µmol/L (both *p* > 0.05).

There was no significant change in MTS assay and cell cycle after EGCG treatment at 1 and 10 µmol/L for 24 and 48 h (Figure 1). With the treatment of EGCG, the viability of hBMSCs was not affected by EGCG at both 1 and 10 µmol/L (both *p* > 0.05). The experiments were repeated at least three times and showed similar effects.

### 2.2. mRNA Expression

**Figure 2:**
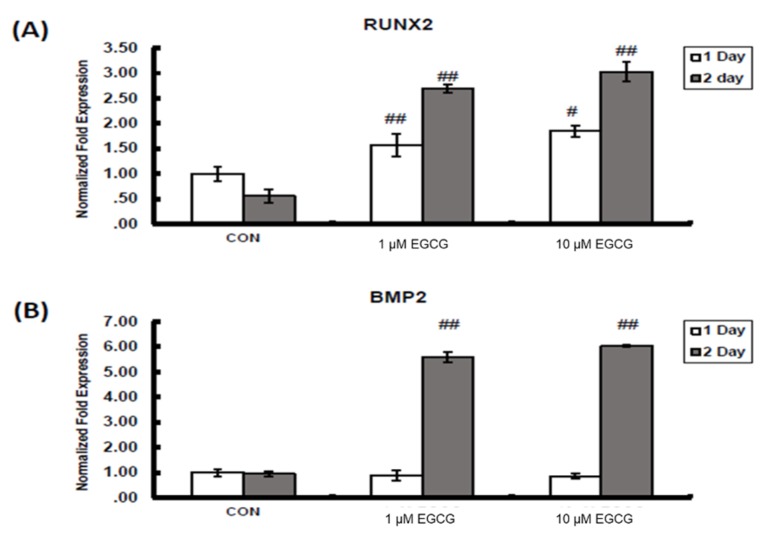

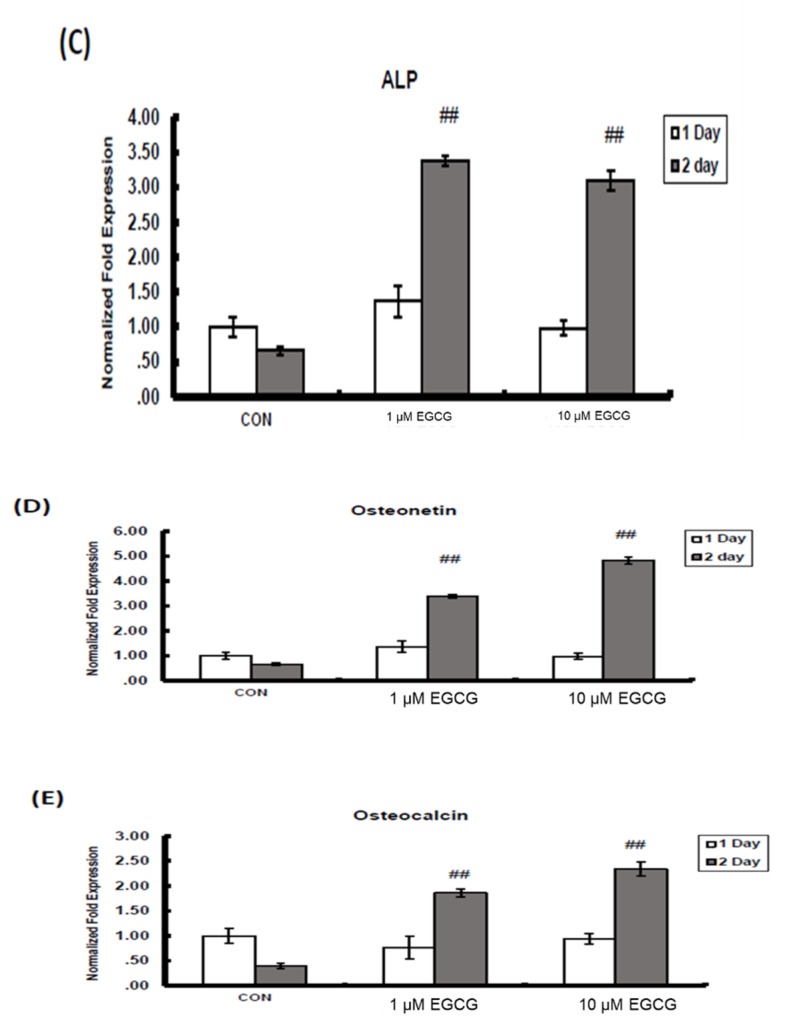
The mRNA expression of osteogenic marker genes. The mRNA expression of Runx2 (**A**), bone morphogenetic protein 2 (BMP2) (**B**), ALP (**C**), osteonectin (**D**) and osteocalcin (**E**) increased significantly after EGCG treatment for 24 and 48 h, at concentrations of both 1 and 10 µmol/L. The expression of BMP2, Runx2, alkaline phosphatase (ALP), osteonectin and osteocalcin were quantified by real-time PCR. There were significant changes in all genes after treatment for 48 h but not 24 h, except in the case of Runx2. In Runx2, mRNA expression increased 57% (*p* < 0.01) and 85% (*p* < 0.05) with 1 and 10 µmol/L, respectively, at 24 h and 169% (*p* < 0.01) and 203% (*p* < 0.01) with 1 and 10 µmol/L, respectively, at 48 h. In BMP2, mRNA expression increased 459% (*p* < 0.01) and 502% (*p* < 0.01) with 1 and 10 µmol/L, respectively. ALP mRNA expression was enhanced 239% (*p* < 0.01) and 210% (*p* < 0.01) at concentrations of 1 and 10 µmol/L of EGCG, respectively. The mRNA expression in osteonectin was amplified 239% (*p* < 0.01) and 383% (*p* < 0.01) after EGCG treatment at concentrations of 1 and 10 µmol/L, respectively. The mRNA expression in osteocalcin was amplified 86% (*p* < 0.01) and 134% (*p* < 0.01) after EGCG treatment at concentrations of 1 and 10 µmol/L, respectively. ^#^ *p* < 0.05, ^##^ *p* < 0.01.

The mRNA expression of osteogenic marker genes, including runt-related transcription factor 2 (Runx2), bone morphogenetic protein 2 (BMP2), alkaline phosphatase (ALP), osteonectin and osteocalcin, increased significantly after EGCG treatment for 24 and 48 h at both the concentrations of 1 and 10 µmol/L. The expression of BMP2, Runx2, ALP, osteonectin and osteocalcin were quantified by real-time PCR. There were significant changes in all genes after treatment for 48 h but not 24 h except in the case of Runx2. In Runx2, mRNA expression increased 57% (*p* < 0.01) and 85% (*p* < 0.05) with 1 and 10 µmol/L, respectively, at 24 h, and 169% (*p* < 0.01) and 203% (*p* < 0.01) with 1 and 10 µmol/L, respectively, at 48 h. In BMP2, mRNA expression increased 459% (*p* < 0.01) and 502% (*p* < 0.01) with 1 and 10 µmol/L, respectively. ALP mRNA expression was enhanced 239% (*p* < 0.01) and 210% (*p* < 0.01) at concentrations of 1 and 10 µmol/L of EGCG, respectively. The mRNA expression in osteonectin was amplified 239% (*p* < 0.01) and 383% (*p* < 0.01) after EGCG treatment at concentrations of 1 and 10 µmol/L, respectively. The mRNA expression in osteocalcin was amplified 86% (*p* < 0.01) and 134% (*p* < 0.01) after EGCG treatment at concentrations of 1 and 10 µmol/L (Figure 2), respectively. The experiments were repeated at least three times and showed similar effects.

### 2.3. Alkaline Phosphatase Activity Assay

**Figure 3:**
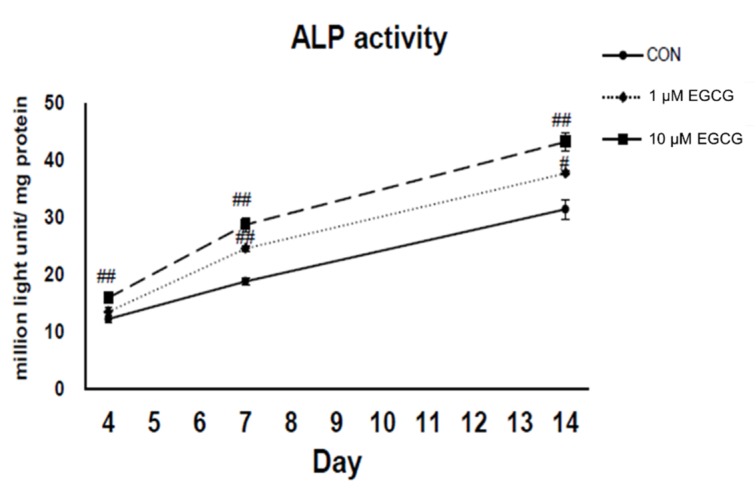
Concentration and time response of ALP activity upregulation by EGCG. In comparison to control cultures, the ALP activities of EGCG (1 and 10 µmol/L)-treated cultures increased by 11% and 30% (*p* < 0.05) on the 4th day, 30% (*p* < 0.01) and 52% (*p* < 0.01) on the 7th day, and 20% (*p* < 0.05) and 37% (*p* < 0.05) on the 14th day, respectively. ^#^ *p* < 0.05, ^##^ *p* < 0.01.

In comparison to control cultures, the ALP activities of EGCG (1 and 10 µmol/L)-treated cultures were increased by 11% and 30% (*p* < 0.05) on the 4th day, by 30% (*p* < 0.01) and 52% (*p* < 0.01) on the 7th day, and 20% (*p* < 0.05) and 37% (*p* < 0.05) on the 14th day, respectively (Figure 3). The experiments were repeated at least three times and showed similar effects.

### 2.4. Mineralization Assay: Alizarin Red S Staining

**Figure 4:**
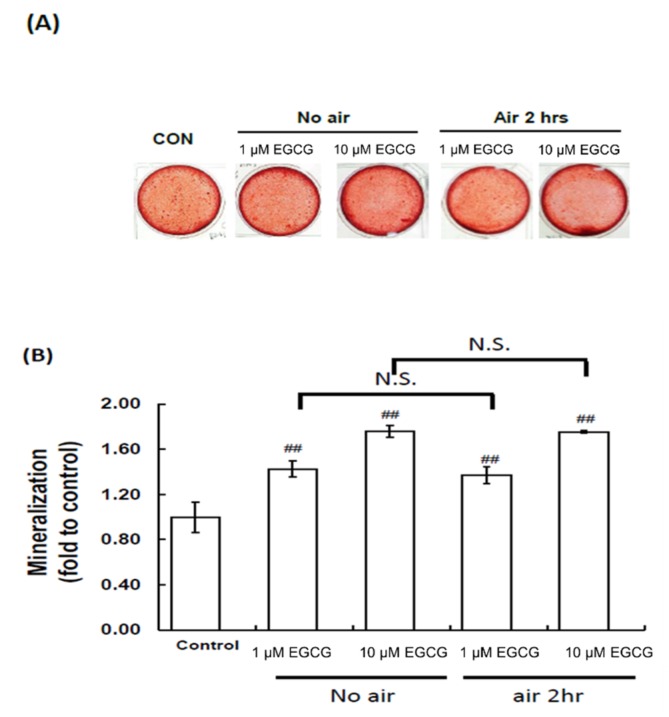
Effects of EGCG on hBMSC mineralization (**A**)–(**B**). Different concentrations of EGCG, 1 and 10 µmol/L, increased mineralization by 43% (*p* < 0.01) and 76% (*p* < 0.01), with respect to the control, respectively. With antioxidant ability evaluated as playing an important role in enhancing mineralization of EGCG in hBMSCs, EGCG was combined with air with O_2_ to deplete its antioxidant ability. After EGCG was combined with air, it was still able to increase mineralization by 37% (*p* < 0.01) and 75% (*p* < 0.01), with respect to the control, respectively. There was no difference between fresh EGCG and EGCG in air, which indicated that antioxidant ability did not play an important role in enhancing mineralization. ^##^ *p* < 0.01 compared with CON.

The cytological results of hBMSC cultures were convincingly positive when stained by Alizarin Red S at the end of the second week with EGCG treatments of 1 or 10 µmol/L (Figure 4). Two different concentrations of EGCG, 1 and 10 µmol/L, increased mineralization by 43% (*p* < 0.01) and 76% (*p* < 0.01), with respect to the control, respectively. With evaluation showing that antioxidant ability played an important role in enhancing mineralization of EGCG in hBMSCs, the EGCG was combined with air to deplete its antioxidant ability. After combining EGCG with air, the EGCG still increased mineralization by 37% (*p* < 0.01) and 75% (*p* < 0.01), with respect to the control, respectively. There was no difference between fresh EGCG and EGCG in air, which indicated that antioxidant ability did not play an important role in enhancing mineralization.

## 3. Discussion

We previously found the double effects of EGCG. EGCG promoted osteogenic differentiation of murine BMSCs at the concentrations of 1 and, in particular, 10 µmol/L [12]. In this study, we found that EGCG can also enhance osteogenic differentiation of hBMSCs in a dose-dependent manner. The effects of EGCG were similar to those on murine BMSCs: enhancement of the expression of osteogenic-related genes, including Runx2, BMP2, ALP, osteonectin and osteocalcin; enhancement of ALP activity; and, eventually, enhancement of mineralization. The higher concentration of 10 µmol/L showed better osteogenic effects than the lower concentration of 1 µmol/L. Moreover, osteogenic effects were not related to its antioxidant activity.

Antioxidant properties of phenolic compounds are important for their beneficial actions, since they can act as scavengers of reactive oxygen species (ROS) [16], but also due to their interaction with intracellular signaling cascades, such as phosphatidylinositol-4,5-bisphosphate 3-kinase (PI3K), protein kinase B (PKB)/Akt, tyrosine kinases, protein kinase C (PKC), and mitogen-activated protein kinases (MAPKs) [17,18,19,20]. The bone anabolic effect exerted by polyphenols may involve different signaling pathways, such as Wnt/b-catenin [21], insulin-like growth factor (IGF1) [22], BMPs [23], Runx2 [12,24] and osterix [12,25].

BMPs are known to play pivotal roles in both cartilage and bone formation [26,27]. BMP2 induces the differentiation of mesenchymal cells into osteoblast precursors, and promotes the maturation of osteoblasts by increasing the expression of Runx2 and osteoblast marker genes [28], and this effect was sufficient to induce optimal matrix mineralization independently of changes in cell growth and type 1 collagen expression [29]. In our previous in vivo study, we found that intraperitoneal EGCG at a dose of 3.4 mg/kg/day with estimated serum concentration of 10 µmol/L for three months can mitigate bone loss and improve bone microarchitecture in ovariectomy-induced osteopenic rats. EGCG-enhanced BMP2 synthesis in bone may stimulate bone formation, and thus contribute to the attenuation of bone loss in ovariectomized rats. The increase of BMP2 expression may contribute to this effect [23]. We further found local EGCG of 40 µL at the concentration of 10 µmol/L at femoral defects can enhance de novo bone formation by increasing bone volume and subsequently improve mechanical properties, including maximum load, break point, stiffness, area under the maximum load curve, area under the break point curve and ultimate stress. Local EGCG may enhance bone defect healing via de novo bone formation of BMP-2, at least in part [30]. In this study, we also found EGCG enhances the mRNA expression of BMP2 and subsequent osteogenic-related genes. BMP2 may play an important role of EGCG in inducing hBMSCs toward osteogenic differentiation. The detailed molecular mechanisms of the signaling pathways involved in the osteogenic effect of EGCG in hBMSCs require further study.

ROS can damage DNA, protein, and lipids. Normal cellular metabolism and environmental stimuli may produce high amounts of ROS and lead to oxidative stress that perturbs the normal redox balance. Excessive oxidative stress decreases osteoblast numbers via nuclear transcription factor κB (NF-κB) signaling pathways [31], and suppresses bone formation rate via Wnt/β-catenin signaling pathways [32]. EGCG is a potent antioxidant. EGCG improves the survival of osteoblasts via suppression of tumor necrosis factor-alpha (TNF-alpha) and interleukin-6 (IL-6) production [33]. EGCG at concentrations of 1–5 µmol/L increases ALP activity and increases in the number and area of mineralized bone nodules as assessed by both von Kossa and Alizarin Red staining in a dose-dependent manner in SaOS-2 human osteoblast (HOB)-like cells [34]. EGCG also increases osteoblastic activity through Wnt signaling pathways, which control bone development and bone mass acquisition [35]. In this study, the aim was to determine whether the antioxidant ability of EGCG played an important role in enhancing osteogenesis. The bioavailability of EGCG is relatively low due to its short half-life and instability by nature. The half-life of EGCG is also short in vivo, ranging from 1.87 to 4.58 h [36]. We found that both fresh EGCG and EGCG combined with air for 2 h possessed the same ability to increase mineralization of hBMSCs. Loss of antioxidant ability of EGCG can still induce hBMSCs toward osteogenic differentiation. Catechin enhanced osteogenic differentiation of immortalized hBMSCs by increasing the level and activity of protein phosphatases 2A (PP2A) that dephosphorylates ERK kinase (MEK) and ERK [37]. EGCG has been shown to induce AMPK activation through an indirect mechanism, by increasing cellular AMP levels [38], while AMPK activation in osteoblasts has been shown to be important for bone nodule formation and maintenance of bone mass [39]. EGCG, at 25 µmol/L, increases ALP activity through activating β-catenin [13,40]. In addition to increased BMP2 expression, EGCG may induce osteogenic differentiation of hBMSCs via the above mechanisms. One recent review paper revealed the binding interaction between EGCG and protein, including fibronectin, matrix metallopeptidase-2 (MMP-2), matrix metallopeptidase-9 (MMP9), vimentin, heat shock protein 90, glucose-regulated protein 78 (GRP78), insulin-like growth factor 1 receptor (IGF1R) and TNF receptor-associated factor 6 (TRAF6) [41]. The receptor requires further study.

A previous in vitro study found EGCG groups, particularly at 5 μM, upregulated BMP-2 expression in hBMSCs [15]. Another study found EGCG can counteract the H_2_O_2_-induced adverse effect on the osteogenic differentiation in hBMSCs. After EGCG treatment, expressions of β-catenin and cyclin D1were upregulated, suggesting that the Wnt pathway was involved in the effects of EGCG on the osteogenic differentiation of hBMSCs [13]. Another study indicated that EGCG itself had little effect on the osteogenic differentiation of MSCs; however, EGCG was able to enhance osteogenesis in the presence of osteoinductive agents through the upregulation of BMP2 expression. It indicated that treatment with EGCG was dependent on other osteogenic inducers [14]. However, whether loss of antioxidant ability of EGCG can enhance osteogenic differentiation of hBMSCs was not studied. In this study, we validated that antioxidant activity of EGCG did not play an important role in osteogenic effects.

Drinking one cup of green tea could lead to a level of EGCG of 1 μmol/L in circulation [5,42]. An oral dose of 1600 mg EGCG can achieve a maximum human plasma level of 7.6 μmol/L under fasting conditions [36]. In this study, the effective concentration of EGCG to enhance osteogenic differentiation of hBMSCs is 1–10 μmol/L. The effective concentration can be easily achieved in daily tea consumption.

In conclusion, EGCG can increase mRNA expression of BMP2 and subsequent osteogenic-related genes, including Runx2, ALP, osteonectin, and osteocalcin. EGCG further increased ALP activity and mineralization. Loss of antioxidant activity can still enhance mineralization of hBMSCs.

## 4. Materials and Methods

### 4.1. Culture of BMSCs

After obtaining informed consent from all patients and approval from the hospital ethics committee (KMU-IRB-970267), leftover bone marrow tissue was acquired from six patients aged 19–40 undergoing orthopedic surgery. We have previously reported the detailed procedures of the isolation and characterization of BMSCs [43,44,45]. Generally, bone marrow samples (5 mL) obtained by tapping from the iliac crest were mixed with 25 mL Dulbecco’s Modified Eagle’s medium (DMEM) and centrifuged at 12,000 rpm for 5 min. After removal of the supernatant, the cell pellet was mixed with DMEM and Percoll (70% in Phosphate-buffered saline), followed by centrifugation at 1560 rpm for 15 min. The medium was changed every 2–3 days.

### 4.2. Catechin Treatment

The EGCG powder was stored at 4 °C. Before the experiments, EGCG was dissolved in dimethyl sulfoxide (DMSO) with a concentration of 10 mmol/L and kept at −20 °C for the remaining experiments. The EGCG stock was diluted with culture medium immediately prior to treatment. Cells were treated by EGCG with concentrations of 1 µmol/L and 10 µmol/L. Accordingly, the concentration of DMSO was less than 0.1% in the experiments. The cultured medium was changed every other day. In the experiments examining mRNA expressions of osteogenic marker genes, the BMSCs were treated with EGCG for 48 h. For the ALP activity assay, cells were harvested at 4, 7 and 14 days after treatment. For the MTS assay, cells were collected after 24 and 48 h treatment. In the mineralization assay, Alizarin Red S staining was performed 2 weeks after treatment with EGCG for 1 week and then cultured in medium for another 1 week. The experiments were repeated at least 3 times.

### 4.3. Real-Time PCR

The mRNA level of genes related to osteogenesis, including Runx2, BMP-2, ALP, osteonectin and osteocalcin, were quantitated by real-time PCR using an iQ5 Real-Time PCR Detection System (Bio-Rad Laboratories, Hercules, CA, USA). In each assay, 1 μg total RNA was treated with 2U DNase I (Ambion, Carlsbad, CA, USA) and reverse-transcribed by Clontech RT-for-PCR kit (BD Biosciences, San Jose, CA, USA) using oligo dT as primers. Real-time PCR reaction mixtures were prepared with iQ SYBR Green Supermix (Bio-Rad Laboratories, Hercules, CA, USA). PCR primer sequences are listed in Table 1, with primer specificity confirmed on the NCBI Primer-BLAST website. Real-time PCR was performed with cDNAs from at least 3 independent experiments. Melting curve analysis was performed for each reaction to ensure a single peak. Amplicons were visualized with electrophoresis on a 1.4% agarose gel to ensure the presence of a single amplicon. Fold changes (x-fold) in gene expression level were calculated by the 2^−ΔΔct^ method [46]. Analysis of variance was performed as in previous studies using Excel 2003 software (Microsoft Corp, Cupertino, CA, USA) [45].

### 4.4. MTS Assay

Briefly, the mitochondria activities of the hBMSCs cultured in wells were detected by the conversion of MTS to formazan as previously described [47,48,49], and the quantity of formazan product released into the medium, which is directly proportional to the number of living cells in culture, can be measured by absorbance at 490 nm [50]. At the indicated time interval, freshly prepared MTS reaction mixture diluted in standard medium at 1:5 (MTS: medium) volume ratio was added to the wells containing the cells and then incubated at 37 °C under 5% CO_2_ for an additional 4 h. After the additional incubation, 100 μL of the converted MTS released into medium from each well was transferred to 96-well plates and the absorbance at 490 nm was recorded with a microplate reader (PathTech) using KC junior software [49].

### 4.5. ALP Activity Assay

The elevation of ALP activities of BMSCs shows that those cells were undergoing osteogenic terminal differentiation. Cells were seeded at 1 × 10^4^ cells per cm^2^ in a 6-well plate in the presence of 10 mmol/L beta-glycerophosphate. Cells were cultured for 4, 7 and 14 days, media with or without EGCG, and were changed every other day. BMSCs were harvested and washed twice with Ca^2+^, Mg^2+^, and NaHCO3 free-PBS. Cell lysate 100 μL was assayed for ALP activity by chemiluminescent method (Tropix Inc., Applied Biosystems, Branchburg, NY, USA). Total protein was determined using a Bio-Rad protein assay kit. The specific activity of ALP was expressed as light unit/mg protein.

### 4.6. Mineralization Assay: Alizarin Red S Staining

Alizarin Red S staining was used to determine the level of extracellular matrix (ECM) calcification 7 days after osteogenic induction. Cells were fixed with 10% formalin-saline at room temperature for 10 min. After washing once with ddH2O, 200 mL Alizarin Red S (Santa Cruz Biotechnology, Dallas, TX, USA) solution (1% in ddH_2_O, pH 4.2) was added to each well of a 48-well plate. The staining solution was removed 10 min later, and each well was washed with H_2_O. The fixed and stained plates were then air-dried at room temperature. The amount of mineralization was determined by dissolving the cell-bound Alizarin Red S in 10% acetic acid and then was quantified spectrophotometrically at 415 nm [12,51].

### 4.7. Statistical Analysis

All data are presented as mean ± standard error. Comparisons of data were analyzed by one-way analysis of variance (ANOVA), and multiple comparisons were performed by Scheffe’s post hoc test (SPSS 10.1 Inc., Chicago, IL, USA). *p <* 0.05 was considered statistically significant.

## Figures and Tables

**Table 1:** Primer sequences and cycling conditions for real-time PCR.

Gene	Primers Sequence (Forward and Reverse)	Annealing Temperature (°C)
GAPDH	Forward: TCTCCTCTGACTTCAACAGCGAC	61
	Reverse: CCCTGTTGCTGTAGCCAAATTC	
RUNX2	Forward: AGA TGGGACTGTGGTTACTG	58
	Reverse: GTAGCTACTTGGGGAGGATT	
BMP2	Forward: GGAATGACTGGATTGTGGCT	64
	Reverse: TGAGTTCTGTCGGGACACAG	
ALP	Forward: CCTCCTCGGAAGACACTCTG	64
	Reverse: GCAGTGAAGGGCTTCTTGTC	
Type I collagen	Forward: GGCTCCTGCTCCTCTTAG	61
	Reverse: CAGTTCTTGGTCTCGTCAC	
Osteocalcin	Forward: GTGCAGAGTCCAGCAAAGGT	61
	Reverse: CGATAGGCCTCCTGAAAGC	
Osteonectin	Forward: GTGCAGAGGAAACCGAAGAG-3′	61
	Reverse: TCATTGCTGCACACCTTCTC-3′	
Cycling conditions	Denature: 95 °C for 30 s, 95 °C for 4 min, followed by 35 cycles of 95 °C for 10 s, 58–64 °C (shown in column of Annealing Temp.) for 15 s and 72 °C for 15 s	
